# Discovery and Preclinical Characterization of Novel Small Molecule TRK and ROS1 Tyrosine Kinase Inhibitors for the Treatment of Cancer and Inflammation

**DOI:** 10.1371/journal.pone.0083380

**Published:** 2013-12-26

**Authors:** Ramesh Narayanan, Muralimohan Yepuru, Christopher C. Coss, Zhongzhi Wu, Matthew N. Bauler, Christina M. Barrett, Michael L. Mohler, Yun Wang, Juhyun Kim, Linda M. Snyder, Yali He, Nelson Levy, Duane D. Miller, James T. Dalton

**Affiliations:** 1 Preclinical Research and Development, GTx, Inc., Memphis, Tennessee, United States of America; 2 Chembridge Research Laboratory, San Diego, California, United States of America; Florida International University, United States of America

## Abstract

Receptor tyrosine kinases (RTKs), in response to their growth factor ligands, phosphorylate and activate downstream signals important for physiological development and pathological transformation. Increased expression, activating mutations and rearrangement fusions of RTKs lead to cancer, inflammation, pain, neurodegenerative diseases, and other disorders. Activation or over-expression of ALK, ROS1, TRK (A, B, and C), and RET are associated with oncogenic phenotypes of their respective tissues, making them attractive therapeutic targets. Cancer cDNA array studies demonstrated over-expression of TRK-A and ROS1 in a variety of cancers, compared to their respective normal tissue controls. We synthesized a library of small molecules that inhibit the above indicated RTKs with picomolar to nanomolar potency. The lead molecule GTx-186 inhibited RTK-dependent cancer cell and tumor growth. *In vitro* and *in vivo* growth of TRK-A-dependent IMR-32 neuroblastoma cells and ROS1-overexpressing NIH3T3 cells were inhibited by GTx-186. GTx-186 also inhibited inflammatory signals mediated by NFκB, AP-1, and TRK-A and potently reduced atopic dermatitis and air-pouch inflammation in mice and rats. Moreover, GTx-186 effectively inhibited ALK phosphorylation and ALK-dependent cancer cell growth. Collectively, the RTK inhibitor GTx-186 has a unique kinase profile with potential to treat cancer, inflammation, and neuropathic pain.

## Introduction

The receptor tyrosine kinase (RTK) family is comprised of 58 transmembrane proteins that regulate many cell functions including proliferation, migration, and cell cycle progression [1]. Increased expression, activating mutations, fusion rearrangements, or coactivation of these proto-oncogenes promote oncogenic transformation of their respective tissues [2], [3]. Due to their functional importance, RTKs have evolved as therapeutic targets for the treatment of cancer, inflammation, pain, neurodegenerative diseases, and others [4]. Discovery efforts to develop small molecule inhibitors or antibodies of RTKs have exponentially increased in the last 10–15 years. Since the discovery of BCR-Abl rearrangement and its inhibitor imatinib, other RTK inhibitors, such as crizotinib (ALK inhibitor), afatinib (EGFR inhibitor), and lenvatinib (VEGFR inhibitor), have been developed for oncology indications [5]–[7].

Tropomyosin-related kinase (TRK) is a family of three RTKs (TRK-A, TRK-B, and TRK-C) regulating several signaling pathways that are important for survival and differentiation of neurons [8], [9]. In addition to their critical function in neurons, they and their ligands (nerve growth factor (NGF), brain derived growth factor (BDNF), and neurotrophins, respectively) are important for non-neuronal cell growth and survival. Increased expression and activation of TRK-A are observed in neuroblastoma, breast cancer, psoriasis, and neuropathic pain, to name a few diseases resulting from TRK-A dysfunction [10]–[12]. Though oncogenic fusions of TRK-A have not been identified to date, its over-expression is sufficient to increase proliferation and invasion of cells. While NGF antibodies are in clinical trials for pain, K252a, the only small molecule TRK-A inhibitor in the clinic, is currently under evaluation for the treatment of psoriasis [13], [14].

ROS1 is a proto-oncogene that belongs to the same phylogenetic branch as TRK-A [15]. Unlike TRK-A, activation of ROS1 typically occurs when it is fused to oncogenic fusion partners such as fused in glioblastoma (FIG) and solute carrier family 34 member 2 (SLC34A2) [2], [16]. ROS1 has been demonstrated to be over-expressed in glioblastoma, cholangiocarcinoma, lung cancer, and others [17]–[19]. Increased expression of ROS1 in various cancers has prompted the development of selective inhibitors. Crizotinib, developed for ALK- positive lung cancer, also inhibits ROS1 and is in a clinical trial for ROS1- positive lung cancer.

The selective pressure applied by continuous RTK inhibition results in the emergence of different clonal populations of cancer cells with acquired resistance and often accelerated proliferation [20], [21]. Escape mechanisms utilized by cancer cells to overcome RTK inhibition include mutations, oncogenic fusion, and activation of secondary kinases and signaling pathways. Hence, development of second and third generation RTK inhibitors is imperative to treat the resistant phenotypes that will inevitably arise from first generation therapy. Developing inhibitors with distinct pharmacophores and unique kinase inhibitory profiles will provide necessary alternate strategies to overcome resistance.

There are a few studies showing ROS1 or TRK-A overexpression in cancers, but individual or combined expression of ROS1 and TRK-A in a broad range of cancers was thus far poorly characterized. Using 381 cDNA samples from 22 cancers, we demonstrate over-expression of TRK-A and ROS1 in cancers that were not previously described. TRK-A is over-expressed in 100% of the pheochromocytoma and the majority of other cancer samples analyzed. GTx-186, a novel RTK inhibitor with unique kinase inhibitory profile, inhibits the TRK family, ROS1, ALK, and RET kinases at picomolar to low nanomolar IC_50_ values. GTx-186 efficiently inhibited cancers driven by TRK-A and ROS1 expression and was also exceptional in overcoming inflammatory diseases such as dermatitis. *In vitro* studies demonstrated that GTx-186 also inhibits neuropathic pain signaling mediated by TRK-A ligand, NGF, making GTx-186 a valuable tool in the armamentarium to combat cancer, inflammation, and pain.

## Materials and Methods

### Reagents

All antibodies were procured from Cell Signaling (Danvers, MA). Realtime PCR reagents and TaqMan primers and probes were obtained from Life Technologies (Carlsbad, CA). The rat cytokine array-2 was obtained from Ray Biotech (Norcross, GA). Cancer survey cDNA array (CSRT 103) was from Origene (Rockville, MD). Human recombinant NGF 2.5 s was from Millipore (Billerica, MA) and dexamethasone was obtained from LKT Labs (St. Paul, MN). Tumor necrosis factor α (TNF-α) and lipopolysaccharide (LPS) were procured from R&D Systems (Minneapolis, MN). Croton oil, carrageenan, and phorbol myristate acetate (PMA) were obtained from Sigma (St. Louis, MO). Indomethacin was from Cayman Chemicals (Ann Arbor, MI). TNF-α ELISA kit was procured from Thermo Scientific (Norcross, GA). GTx-186 (S-isomer) and crizotinib (racemate) were synthesized by GTx chemists and were characterized by standard analytical techniques. All other reagents used were analytical grade.

### Kinase Activity Assay

Compounds to be tested were dissolved in 100% DMSO in a range of concentrations from 10^−8^ to 10^−3^ M, then diluted with kinase assay Buffer (50 mM HEPES, pH 7.5, 1 mM EGTA, 10 mM MgCl_2_, 0.01% Tween-20, and 2 mM DTT, added fresh) to 4X the final concentration. The final curve included 11 concentrations (10^−11^ to 10^−5^ M). Kinase (Invitrogen) and ATP (Sigma) concentrations necessary for optimal activity were determined in separate experiments (Table S1). The concentration of kinase that resulted in maximal activity and the concentration of ATP that showed 50% maximal stimulation (EC_50_) were chosen for enzyme inhibitor experiments.

Kinase assays were performed in a final volume of 10 µl in 384-well plates, with 2.5 µl of test compound in triplicate at each concentration, 2.5 µl of kinase, and 5 µl of ATP and peptide substrate (LANCE® *Ultra* U*light*™ poly-GT, PerkinElmer) mix. Reactions were incubated at room temperature (RT), in the dark, for 30–120 min. Following incubation, reactions were stopped with the addition of 40 mM EDTA in 1X LANCE® buffer (PerkinElmer) (5 µl), and incubated at RT for 5 min. LANCE® Eu-W1024 anti-phosphotyrosine antibody PT66 (5 µl) in 1X LANCE® buffer was added to the wells at a final concentration of 1.25 nM and incubated for 1 h at RT. The relative amount of phosphorylated substrate was measured with VICTOR™ Multilabel Plate Reader using the LANCE™ protocol for time-resolved fluorescence resonance energy transfer. The concentration of test compound required to decrease the fluorescence signal (665 nm) by 50% (IC_50_) value, was determined by non-linear regression with Sigma Plot® and the standard four parameter logistic curve.

### Cell Culture

IMR-32 and NIH3T3 were obtained from ATCC (Manassas, VA) and were grown according to the instructions provided. The cell lines authenticated by the provider were cultured for less than 6 months after resuscitation in the laboratory. For growth factor-induced gene expression experiments, cells were plated in 96-well plates at 10,000 cells per well in medium supplemented with 1% charcoal stripped FBS (csFBS). Cells were maintained in 1% csFBS for 3 days to reduce basal transcription with medium changed on day 1 and before treatment on day 3. Cell plating and growth conditions for all other experiments are as described in the figure legends.

The lymphoma line U-937 (ATCC Manassas VA) and anaplastic large cell lymphoma lines SUDHL-1 and K-299 (DSMZ, Braunschweig, Germany) were maintained in RPMI-1640 supplemented with 10% fetal bovine serum and 100 U/mL of penicillin/streptomycin. Kelly cells (DSMZ, Braunschweig, Germany) were grown in RPMI-1640 and 10% FBS.

BaF_3_ cells were obtained from DSMZ (Braunschweig, Germany) and maintained in RPMI-1640 supplemented with 10% fetal bovine serum, 10 ng/µL Interleukin-3 (R&D Systems Minneapolis, MN) and 100 U/mL of penicillin/streptomycin. BaF_3_ stable cell lines were maintained in RPMI-1640 supplemented with 10% fetal bovine serum, 100 U/mL of penicillin, and 500 µg/mL G418 sulfate solution (Mediatech).

### Cloning and Stable Cell Line Creation

All plasmid constructs were sequenced to ensure fidelity. FIG-ROS1 (S) [17], synthesized by Genscript (Piscataway, NJ), was cloned into pCMV6 vector and then sub-cloned into pLenti U6 Pgk-puro vector. Stable NIH3T3 cell lines were generated by lentiviral infection of pLenti U6 Pgk-puro-FIG-ROS1 (S) as described earlier [22]. The NPM-ALK fusion was obtained from cDNA amplified from K299 cells and cloned into pCR3.1 (Life Technologies Carlsbad, CA) with EcoRI. EML-4ALK was synthesized by Genscript from reference sequence AB274722 representing the E13:A20 fusion and was also cloned into pCR3.1 with EcoRI. Insert orientation and fidelity were confirmed by DNA sequencing.

### RNA Isolation and Gene Expression

RNA was isolated using the Cells-to-Ct kit and realtime PCR was performed using TaqMan primers and probes on the ABI 7900 (Life Technologies). cDNA array experiments were conducted using TaqMan primers and probes on the ABI 7900 PCR machine.

### Western Blotting

Cells were grown and treated as described in the figure legends. Protein extracts were prepared and the extracts were run using a SDS-PAGE on a 4–20% gradient gel and immunoblotted for the indicated proteins.

### Growth Assay and Cell Cycle Analysis

Adherent cells were plated at 10,000 cells per well in 96-well plates in respective medium. The cells were treated as indicated in the figures and cell viability was measured using sulforhodamine B (SRB) reagent and the optical density measured at 535 nm. For cell cycle analysis, cells were plated in 6 well plates and treated for the indicated time points. Cells were fixed, stained with propidium iodide, and distribution in different phases of cell cycle was evaluated using flow cytometry.

### Migration Assay

Migration assay was performed using platypus migration assay kit (Fisher Scientific). Cells were seeded in 96 well plates and the inserts were removed 24 hrs after seeding. Cells were treated as indicated in the figures and imaged 12 hrs after treatment.

### Cytokine Array

Cytokine arrays were obtained from Ray Biotech (Norcross, GA) and arrays were processed according to manufacturer’s instructions. Briefly, arrays were incubated overnight with equal volume of air pouch exudates, washed, and incubated with secondary antibodies before developing using enhanced chemiluminescence.

### ELISA p-ALK Quantification

K-299 cells were treated with crizotinib or GTx-186 for six hours. Cell lysates were then isolated and protein was extracted using Cell Lysis Buffer (Cell Signaling) containing Protease Inhibitor Cocktail (Roche Diagnostics), PMSF (Sigma Aldrich), and Phosphatase Inhibitors Cocktail (Sigma Aldrich). p-ALK ELISAs (Cell Signaling Technology Beverly, MA) were performed on cell lysates according to the manufacturer’s instructions using 0.01 µg/µl of protein.

### Animal Experiments

All animal protocols were approved by The University of Tennessee Institutional Animal Care and Use Committee. Mice and rats obtained from Harlan (Indianapolis, IN) were housed with five or three animals per cage, respectively, and were allowed free access to water and commercial rodent chow (Harlan Teklad 22/5 rodent diet - 8640). During the course of the study, animals were maintained on a 12 hr light:dark cycle.

### Tumor Xenograft Experiments

Xenograft experiments were performed in nude mice as previously described [23]. Briefly, a mixture of cells suspended in 0.0375 mL RPMI+10% FBS and 0.0625 mL Matrigel was injected s.c. into the hind left flank of each mouse. Once the tumor volume reached 100–200 mm^3^, animals were randomized and treated (GTx-186 was dissolved in 81% PEG-300+18% sterile double deionized water+0.6% 12 N hydrochloric acid; crizotinib was dissolved in 10% DMSO+90% PEG-300) as indicated in the figures. Tumor volume and body weight were measured as indicated in figures. Tumor volume was calculated using the formula length*width*width*0.5236.

### Croton Oil-induced Dermatitis

C57BL/6 mice were treated twice at 16 hrs and 2 hrs before application of acetone or croton oil (20 µl of 10% croton oil in acetone on each inner ear) [24]. Six hours after croton oil application, animals were sacrificed, and ear punches were weighed and stored in RNA later for RNA isolation.

### Air Pouch Inflammation Model

Air was injected in the flanks of Sprague Dawley rats four days (20 mL) and two days (10 mL) before injection of carrageenan (1 mL of 2% carrageenan) in the pouch [25]. Six hours after carrageenan administration, animals were sacrificed, 5 mL saline was injected into the pouch, pouch exudates were collected and the number of leukocytes and macrophages infiltrated into the pouch were counted under a microscope.

Statistical analyses were performed using GraphPad Prism software.

## Results

### TRK-A and ROS1 are Over-expressed in Multiple Cancers

**Figure 1:**
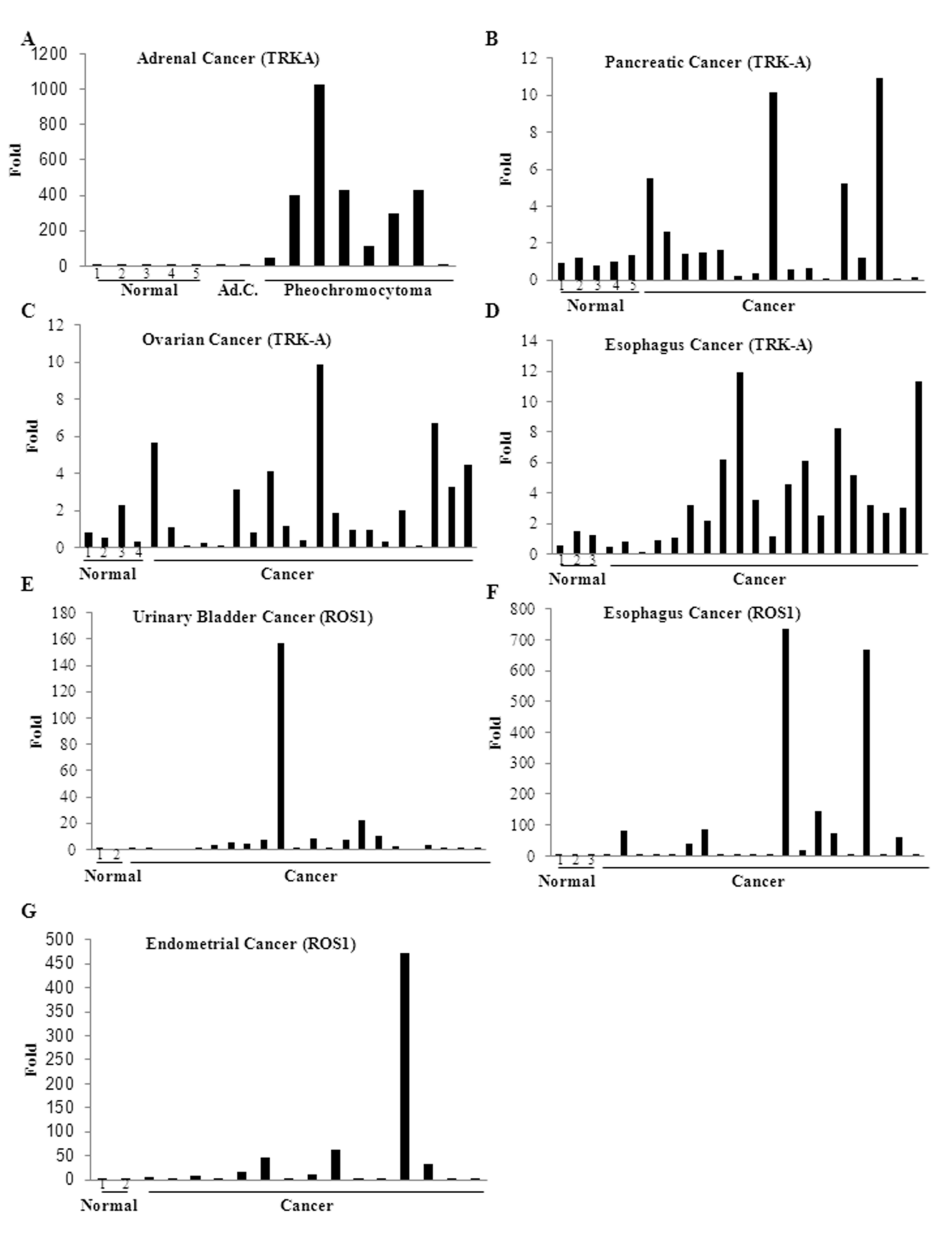
TRK-A and ROS1 are over-expressed in multiple cancers. Expression of TRK-A and ROS1 were quantified by realtime PCR in cDNAs from 381 samples from 22 different cancers and corresponding normal tissues. TRK-A and ROS1 expression were normalized to actin and represented as fold difference from normal non-cancerous samples using ddCt method. Average of normal samples was taken for the ddCt calculation. Ad.C-Adrenal cancer; Normal-cDNA from Non-cancerous tissues. Numbers under samples indicate normal samples.

Isolated studies have identified the over-expression of either TRK-A or ROS1 in neuroblastoma [26], lung cancer [2], glioblastoma [16], and cholangiocarcinoma [17]. Since both these kinases are proto-oncogenes, we speculated that information on combined expression could help to predict additive or synergistic growth of the respective cancer. To determine the TRK-A and ROS1 expression pattern, tissue scan cDNA arrays containing 381 cDNA samples from 22 cancers and adjacent normal tissues were used (Figure 1). Cancers of adrenal (7/10 cancer samples expressed TRK-A), pancreas (5/17 TRK-A), ovary (7/20 TRK-A), esophagus (9/20 TRK-A and ROS1), urinary bladder (6/22 TRK-A), and endometrium (9/17 ROS1) expressed increased levels of TRK-A and/or ROS1, compared to corresponding normal samples. Interestingly, 100% of the pheochromocytoma, a neuro-endocrine adrenal cancer with sympathetic nervous system origin, samples over-expressed TRK-A between 50–1000 fold, compared to normal adrenal tissues. Earlier reports have shown that the pheochromocytoma cell line, PC12, expressed the highest level of TRK-A among numerous *in vitro* cellular systems examined, corroborating these findings [27]. Samples from cancers of the urinary bladder (3 samples) and esophagus (4 samples) expressed both TRK-A and ROS1, which could potentially lead to additive or synergistic proliferation. Cancers of prostate, breast and others failed to express detectable levels of either of the kinases. Since these results are from a small subset of samples, they have to be validated with additional samples.

### In vitro Characterization of GTx-186

GTx186 is an imidazo[4,5-f]isoindole derivative related to previously the disclosed novel RTKI GTx-134 [28]. To leverage the oncology, inflammatory, and nociceptive roles of TRK-A and ROS1 tyrosine kinases, we built a library of small molecules that selectively inhibits the kinases in the phylogenetic branch containing TRK-A and ROS1. GTx-186 selectively inhibited TRK-A, TRK-B, TRK-C, ROS1, ALK, and RET-mediated phosphorylation with IC_50_ values in the picomolar to low nanomolar range (Table 1). On the other hand, GTx-186 inhibited IGF-1R and EGFR-mediated phosphorylation at much higher concentrations (greater than 100–1000 nM), indicating its selectivity towards only a subset of kinases. Since ALK and RET expression in normal tissues are minimal and knockout animals have inconsequential phenotypes [29], [30], cross reactivity with these two kinases are of small concern enabling GTx-186 to treat TRK-A and ROS1-dependent diseases, with minimal risk of unwanted effects.

**Table 1:** Kinase assays to determine the effect of GTx-186 and staurosporine on the activity of the indicated kinases.

Drug	ALK (nM)	EGFR (nM)	TRK-A (nM)	TRK-B (nM)	TRK-C (nM)	ROS1 (nM)	RET (nM)
**Staurosporine**	3.627	33.92	1.307	1.366	0.773	0.306	2.033
**GTx-186**	4.510	6848	1.141	2.064	1.585	0.205	5.766

Values are expressed as IC_50_.

### GTx-186 Inhibits TRK-A-dependent IMR-32 Neuroblastoma Cell and Xenograft Growth

**Figure 2:**
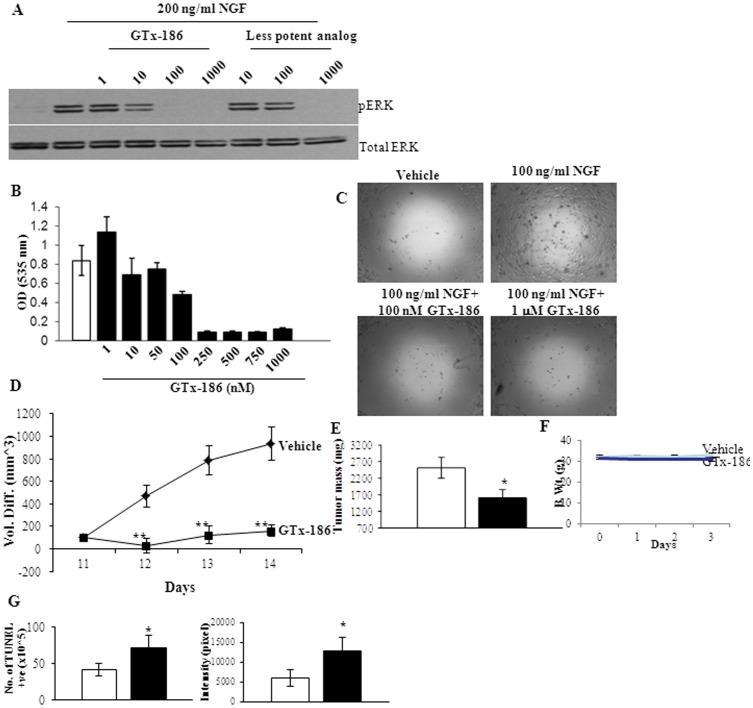
GTx-186 inhibits TRK-A-dependent neuroblastoma cell and tumor xenograft growth. **A**. GTx-186 inhibits NGF-induced ERK (p42/44 MAPK) phosphorylation. IMR-32 neuroblastoma cells were serum starved for 2 days, pre-treated for 2 hrs with the indicated concentrations of GTx-186 and treated with 200 ng/ml NGF for 30 min. Cells were harvested and Western blot performed for phospho-ERK and total-ERK. **B**. GTx-186 inhibits proliferation of IMR-32 cells. IMR-32 cells were plated in growth medium and treated with indicated concentration of GTx-186 for 3 days. Cells were fixed and stained with sulforhodamine B (SRB) and optical density (OD) was measured at 535 nm. **C**. GTx-186 inhibits migration of IMR-32 cells. IMR-32 cells were plated in a platypus migration assay plate and were treated with vehicle, NGF, or combination of NGF and GTx-186. Images were captured under light microscope 12 hrs after treatment. **D–G**. GTx-186 inhibits IMR-32 neuroblastoma xenograft growth. IMR-32 cells were subcutaneously implanted in nude mice (10 million cells/mouse). Once tumors reached 100–200 mm^3^, animals (n = 8) were randomized and treated daily with vehicle or 20 mg/kg/day i.v. GTx-186. Tumor volume (D) and body weight (F) were measured every day for 4 days. The animals were sacrificed, tumors weighed (E), stored in formalin, and processed for TUNEL immunohistochemistry (G-Number of TUNEL positive cells (left panel) and intensity of TUNEL staining (right panel)). Values are expressed as Avg ± S.E. NGF-Nerve Growth Factor; Open bars are vehicle-treated and filled bars are GTx-186-treated samples. *-statistically significant at p<0.05; **-statistically significant at p<0.01.

Since IMR-32 neuroblastoma cells express TRK isoforms and are dependent on TRK-A for their growth [31], we used this line as a model to study the effects of GTx-186 on TRK-A phosphorylation and growth, both *in vitro* and *in vivo*. The highly potent GTx-186 inhibited NGF-induced phosphorylation of p42/44 MAPK, a kinase downstream of TRK-A that mediates proliferation and inflammation in response to NGF [32], at a concentration as low as 10 nM (Figure 2A). On the other hand, a 10-fold less potent analog inhibited NGF-induced p42/44 MAPK phosphorylation only at a concentration greater than 100 nM. Subsequently, IMR-32 cells were treated with increasing concentrations of GTx-186 to determine the effect on cell growth after 72 hours. As shown in Figure 2B, GTx-186 dose-dependently inhibited the growth of IMR-32 cells with an IC_50_ value of approximately 100 nM. GTx-186 also completely inhibited NGF-dependent migration of IMR-32 cells (Figure 2C), indicating that inhibiting TRK-A is both anti-proliferative and anti-invasive. Under identical conditions, GTx-186 had no effect on the growth of SH-SY5Y cells, a neuroblastoma cell line that lack TRK-A [26].

To demonstrate that the anti-proliferative effects of GTx-186 are reproducible *in vivo* in a tumor xenograft model, IMR-32 cells were implanted in nude mice and tumor bearing animals were treated with vehicle or GTx-186 intravenously. Due to the rapid proliferative and highly invasive nature of IMR-32 cells, the tumors grew from 200 mm^3^ to 2000 mm^3^ within 4 days. GTx-186 effectively and significantly reduced the tumor growth (Figure 2D) starting from day 1 until the end of the study, resulting in greater than 80% tumor growth inhibition. These effects were elicited without any visible toxic effects, including any changes in body weight (Figure 2F). GTx-186 also significantly reduced tumor weight (Figure 2E) by more than 50% compared to vehicle-treated animals. In order to determine the mechanism for this anti-tumor effect, formalin fixed tumor tissues were immunohistochemically stained for TUNEL and the number of TUNEL-positive cells and TUNEL staining intensity were evaluated. GTx-186 increased apoptosis of tumor cells by more than two fold compared to vehicle-treated animals as shown by both the number of TUNEL-positive cells and TUNEL-staining intensity (Figure 2G). These effects were elicited with a steady state concentration of approximately 50 nM GTx-186, which is comparable to *in vitro* IC_50_ values.

### GTx-186 Elicits Anti-inflammatory Effects in vitro

**Figure 3:**
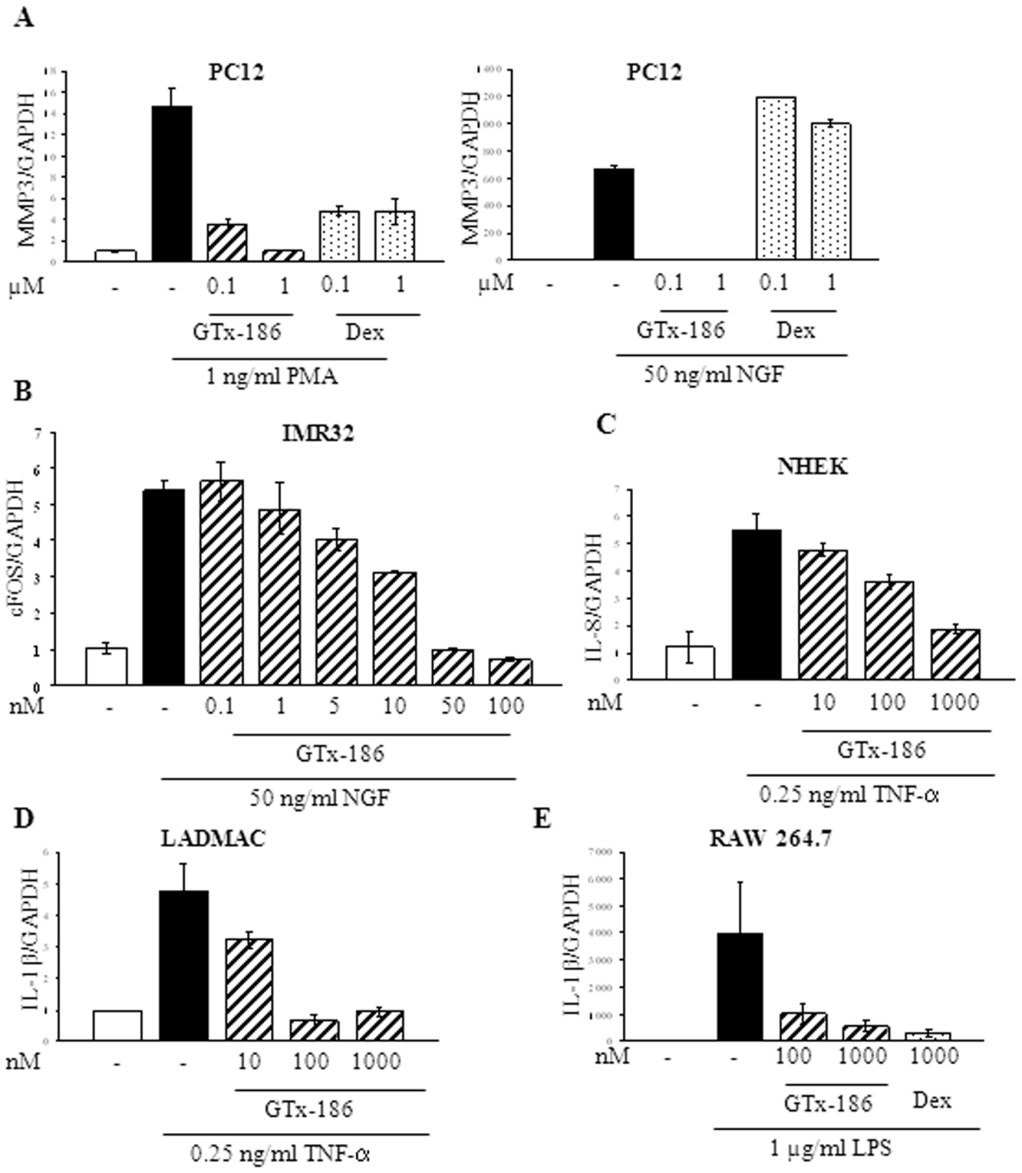
GTx-186 inhibits inflammation *in vitro*. Cells were serum starved for 2 days, pre-treated with indicated concentrations of GTx-186 for 30 min and treated with growth factors or cytokines for 12–16 hrs. RNA was extracted, cDNA synthesized, and expression of inflammatory genes were measured by realtime PCR using Taqman primer and probes. All experiments were performed in triplicate and repeated at least three times. Values are expressed as Avg ± S.E. Dex-Dexamethasone; PMA-Phorbol Myristate Acetate; NGF-Nerve Growth Factor; TNF-α-Tumor Necrosis Factor; LPS-Lipopolysaccharide; MMP3-Matrix Metalloproeinase 3; IL-8-Interleukin 8; PC-12-Pheochromocytoma cells; IMR-32-Neuroblastoma cells; NHEK-Normal Human Epidermal Keratinocytes; LADMAC-Macrophage/Monocytes; RAW-264.7-Mouse macrophage cells.

TRK-A and its ligand NGF are mediators of inflammatory diseases such as dermatitis, psoriasis, and arthritis. NGF is expressed in macrophages and *in vitro*, NGF induces TNF-α synthesis and synergizes with interferon-γ to increase the expression of inflammatory cytokines [33]. These effects may be reversed by TRK-A inhibitors. In addition, NGF and TRK-A have been implicated in a variety of inflammatory and allergic conditions, including asthma and polymorphonuclear leukocyte chemotaxis [34]. To understand the effect of GTx-186 on inflammation induced by various cytokines and growth factors, PC12 cells were pre-treated with GTx-186 or dexamethasone and subsequently treated with PMA or NGF to induce the expression of inflammatory cytokines. While GTx-186 effectively inhibited the expression of MMP-3-, a matrix metalloproteinase, induced by both PMA and NGF, dexamethasone inhibited MMP-3 expression induced only by PMA, but not by NGF (Figure 3A). This demonstrates that NGF and PMA mediate inflammation through distinct pathways, and glucocorticoids, such as dexamethasone, are effective only in non-NGF-mediated inflammation. To determine broader anti-inflammatory effects of GTx-186, if any, IMR-32 cells (Figure 3B), normal human keratinocytes (NHEK; Figure 3C), LADMAC macrophages (Figure 3D), and RAW 264.7 mouse macrophages (Figure 3E) were pre-treated with GTx-186, and inflammatory cytokines were induced by NGF, TNF-α, or LPS. GTx-186 effectively inhibited important inflammatory mediators such as cFos, IL-8, and IL-1β with potency comparable to its effects on kinase activity.

### GTx-186 Inhibits Croton Oil-induced Atopic Dermatitis in Mice

**Figure 4:**
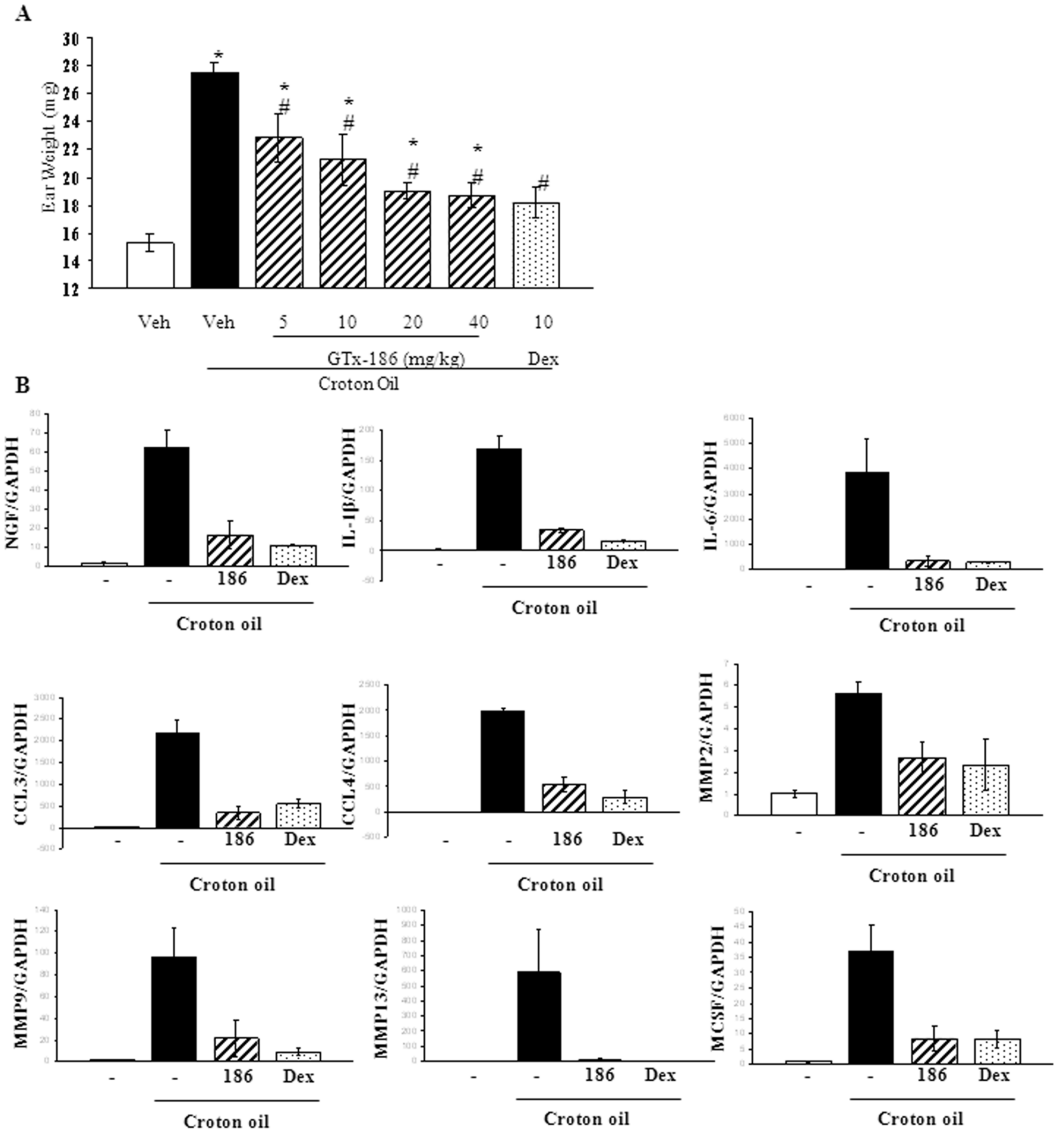
GTx-186 inhibits croton oil-induced atopic dermatitis in mice. A. C57BL/6 mice (20–25 gms; n = 3) were administered twice with the indicated dose of GTx-186 (s.c.) or dexamethasone (s.c.), 16 hrs and 2 hrs, before application of croton oil (20 µl of 10% croton oil in acetone on each inner ear). Six hours after croton oil application, animals were sacrificed, ear punches were weighed and stored for RNA isolation. *indicates significance at P<0.05 from acetone applied vehicle-treated animals. # indicates significance at P<0.05 from croton oil applied vehicle-treated animals. B. RNA was isolated from ear punches of the animals in panel A and expression of indicated genes were measured by realtime PCR and normalized to GAPDH using TaqMan primers and probes. Values are expressed as Avg ± S.E. Dex-dexamethasone; 186-GTx-186; NGF-Nerve Growth Factor; IL-Interleukin; CCL-Chemokine ligand; MMP-Matrix Metalloproteinase; MCSF-Macrophage Colony Stimulating Factor.

Since TRK-A has been implicated in psoriasis [12] and dermatitis [35] and GTx-186 inhibited IL-8, one of the critical cytokines that mediate dermal inflammation [36] (Figure 3C), we evaluated GTx-186 in a croton oil model of atopic dermatitis. Croton oil increases erythema, ear weight, and vascular leakage, a phenotype similar to atopic dermatitis [37]. Application of croton oil increased edema and weight of ears, which were dose dependently reduced by GTx-186 (Figure 4A). RNA from ear punches were isolated and expression of various genes in the inflammatory pathways was measured (Figure 4B). Croton oil increased the expression of all the measured inflammatory pathway genes, including NGF, which were all markedly inhibited by GTx-186 and dexamethasone.

### GTx-186 Inhibits Carrageenan-induced Inflammation in Rat Air Pouch Model

**Figure 5:**
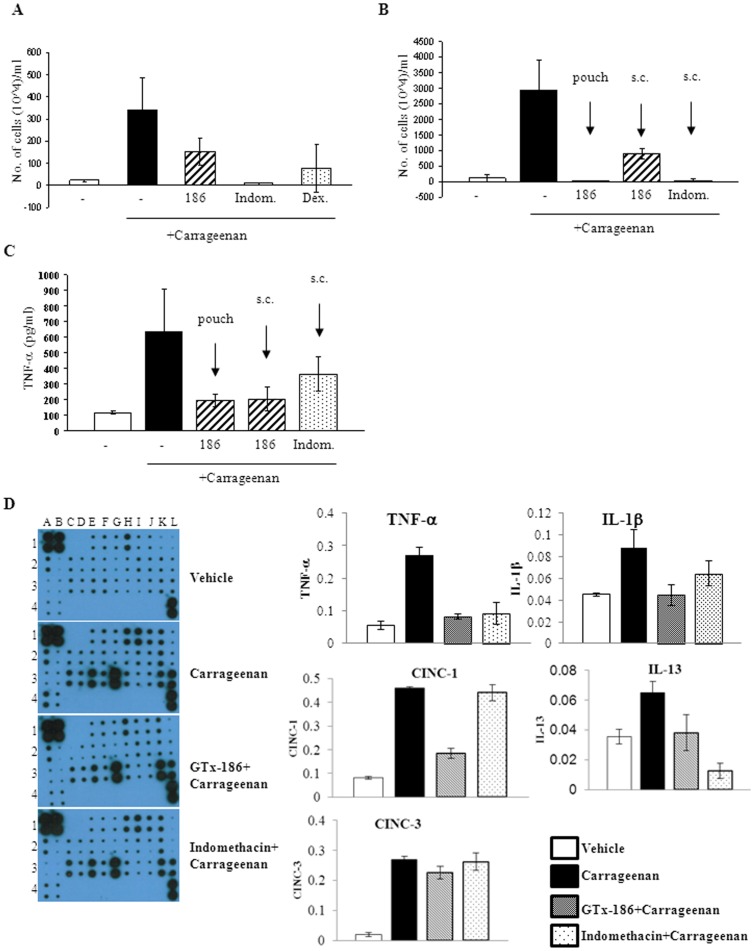
GTx-186 inhibits carrageenan-induced air-pouch inflammation in rats. A–C. A pouch was created subcutaneously in one flank of rats (n = 3) by injecting 20 ml air four days before and 10 ml air two days before treatment. GTx-186 (s.c. in panel A and intra-pouch or s.c. in panel B; 40 mg/kg), dexamethasone (30 mg/kg s.c.), or indomethacin (30 mg/kg s.c.) were administered thrice, 48 hrs, 24 hrs, and 1 hr, before administration of carrageenan (1 ml of 2%). Six hours after carrageenan administration, the animals were sacrificed, 5 ml saline was injected into pouch to flush the pouch fluid and the number of leukocytes and macrophages infiltrated into the pouch were counted under a microscope. TNF-α was measured using an ELISA in the pouch exudates from the experiment in panel B. (C). D. Effect of GTx-186 on inflammatory cytokines in carrageenan-induced air-pouch inflammation was measured in the pouch exudates using a cytokine array. Key cytokines were quantified and expressed as bar graphs on the right. Values are expressed as Avg ± S.E. of n = 3. 186-GTx-186; Indom-Indomethacin; Dex-Dexamethasone; TNF-Tumor Necrosis Factor; IL-Interleukin; CINC-Cytokine-Induced Neutrophil-Chemoattractant protein.

NGF antibodies are clinically successful in treating arthritic pain [38]. However, the potential to treat systemic inflammation in conjunction with pain was not evaluated. Since GTx-186 inhibited inflammatory cytokines in a variety of cell lines, including macrophages, we tested GTx-186 in carrageenan-induced systemic inflammation using an air pouch model. The air pouch model is physiologically and mechanistically comparable to arthritis, and hence this model was used to evaluate GTx-186 [25], [39]. Administration of carrageenan increased leukocyte and macrophage infiltration into air-pouch, compared to saline-treated animals (Figures 5A and 5B). Subcutaneous and intra-pouch administration of GTx-186 significantly reduced leukocyte and macrophage infiltration. Intra-pouch administration of GTx-186 elicited a better response than subcutaneous administration, indicating a positive correlation between drug exposure at the site of inflammation and the anti-inflammatory effects.

Air pouch exudates were subjected to cytokine array to determine the effect of GTx-186 and indomethacin on the global cytokine protein expression (Figure 5D and Table S2). Interestingly, GTx-186 and indomethacin differed in their effects to inhibit cytokines. While both GTx-186 and indomethacin inhibited carrageenan-induced TNF-α, IL-1β, fractalkine, IL-13, and leptin, only GTx-186 inhibited CINC-1, CINC-2, IL-6, and LIX. On the other hand, neither GTx-186 nor indomethacin inhibited carrageenan-induced CINC-3, TIMP-1, PDGFa, MMP-8, and MCP-1, indicating that these cytokines may not be critical mediators of inflammation in the air pouch model.

TNF-α is an important mediator of inflammation in carrageenan-induced acute inflammation in air pouch synovial models. Local levels of TNF-α correlated with the swelling and extent of inflammation, which were reversed by an anti-TNF-α antibody [25]. In the experiments demonstrated in Figures 5C and 5D, TNF-α levels increased significantly after carrageenan administration, which were effectively inhibited by both GTx-186 and indomethacin.

### GTx-186 Inhibits Nociceptive TRK-A Signaling in PC12 Cells

Since TRK-A is one of the primary mediators of neuropathic pain and NGF antibodies are currently in a clinical trial to alleviate pain arising from various etiologies [40], [41], we evaluated the effects of GTx-186 on neurite outgrowth and expression of early genes in the nociceptive pathways. Expression of cFOS and its product FOS are increased rapidly and transiently when rats are exposed to noxious stimuli such as heat or mustard oil [42]. NGF-induced neurite outgrowth in PC12 cells [43] was completely inhibited by 100 nM GTx-186 (Figure S1A) without directly affecting their proliferation (Figure S1B). While GTx-186 dose-dependently inhibited the rapid expression of c-FOS and NGF1A induced by NGF, it failed to inhibit the expression of c-FOS induced by EGF, indicating its specificity to NGF-dependent pathways (Figure S1C).

### GTx-186 Inhibits ROS1 Kinase Phosphorylation and ROS1-dependent Cell Growth

**Figure 6:**
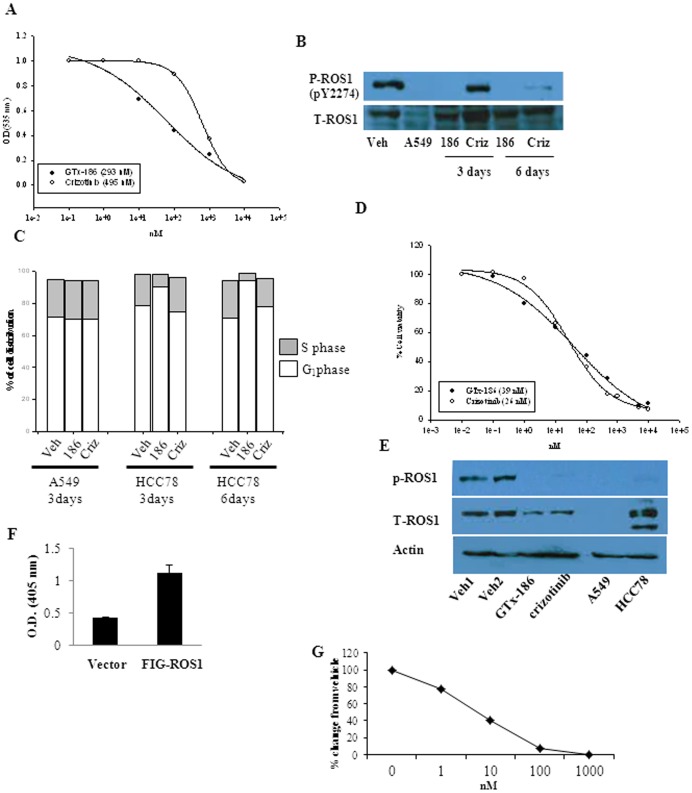
GTx-186 inhibits growth of ROS1-dependent cell lines. A. GTx-186 inhibits the growth of HCC78 lung carcinoma cells. HCC78 lung carcinoma cells were plated in growth medium and treated with indicated concentrations of GTx-186 or crizotinib on days 1 and 6. Cells were fixed after 6 days and stained with sulforhodamine B (SRB) and OD measured at 535 nm. B. GTx-186 inhibits phosphorylation of ROS1. HCC78 cells were plated in growth medium and treated with 100 nM GTx-186 or crizotinib for 3 or 6 days. Protein was extracted and immunoblotted with phospho-ROS1 and total ROS1 antibodies. C. GTx-186 arrests cells in G_1_ phase of the cell cycle. HCC78 or A549 cells were treated for 3 or 6 days with 1 µM GTx-186 or crizotinib and flow cytometry was performed to determine the cell cycle distribution. A549 cells were used as ROS1 negative lung cancer control cells as they do not express ROS1. D and E. GTx-186 inhibits phosphorylation of ROS1 and growth of NIH3T3 cells over-expressing FIG-ROS1. NIH3T3 cells stably transfected with FIG-ROS1 were treated with GTx-186 or crizotinib for 3 days and the growth of cells (D) and phosphorylation (100 nM GTx-186 or crizotinib) of ROS1 (E) were measured by SRB assay and Western blotting, respectively. Cells in panel E were treated with indicated concentrations of GTx-186 and crizotinib. F and G. Over-expression of FIG-ROS1 increases proliferation of Ba/F3 cells. Ba/F3 cells stably transfected with vector or FIG-ROS1 were plated in equal numbers and growth of the cells after 3 days was measured by SRB assay (F). Ba/F3 cells stably expressing FIG-ROS1 were treated with indicated concentrations of GTx-186 for 3 days and growth of cells were measured by sulforhodamine B (SRB) assay. Values are expressed as Avg ± S.E. of n = 3.

ROS1 is another proto-oncogene expressed as a fusion protein in multiple cancers [2], [17], [44]. Since GTx-186 inhibited ROS1 tyrosine kinase potently (Table 1), we evaluated the effect of GTx-186 on the growth of various ROS1-dependent cell lines and also compared the effects with a commercially available known ROS1 inhibitor, crizotinib. GTx-186 and crizotinib inhibited the proliferation of HCC78 lung cancer cells, which harbors SLC34A2:ROS1 fusion and are dependent on ROS1 for growth [2], with an IC_50_ value of 293 nM and 495 nM, respectively (Figure 6A). Concurrently, phosphorylation of ROS1 was examined in HCC78 cells. GTx-186 inhibited ROS1 phosphorylation in 3 days, while crizotinib inhibited only after 6 days (Figure 6B). Similar to the phosphorylation results, GTx-186 was highly effective in arresting cells in G_0_/G_1_ phase of the cell cycle in 3 days, while crizotinib arrested the cells only after 6 days (Figure 6C). Growth of A549 cells, a lung cancer model that does not express ROS1, was not inhibited by GTx-186 or crizotinib.

In order to understand if GTx-186 is effective against ROS1 fused to other partners such as FIG, NIH3T3 cells were stably transfected with FIG-ROS1 (Short) (NIH3T3:FIG-ROS1 (S)). Previous publications indicated that this shorter FIG clone fused to ROS1, expressed in cholangiocarcinoma, is highly proliferative and invasive [17]. Both GTx-186 and crizotinib efficiently inhibited the proliferation of NIH3T3:FIG-ROS1 (S) with IC_50_ values of 39 nM and 26 nM, respectively (Figure 6D). In addition, as demonstrated with HCC78 cells, GTx-186 and crizotinib were extremely effective in inhibiting the phosphorylation of ROS1 fused to FIG (Figure 6E).

Over-expression of proto-oncogenes facilitates the normally IL-3-dependent Ba/F3 cells (murine B cells) to grow in the absence of IL-3 and hence this model was utilized to demonstrate the effects of GTx-186 [45]. Consistent with previous publications, over-expression of FIG-ROS1 (S) in Ba/F3 cells (Ba/F3:FIG-ROS1 (S)) increased its proliferation (Figure 6F), which was efficiently inhibited by GTx-186 (Figure 6G).

**Figure 7:**
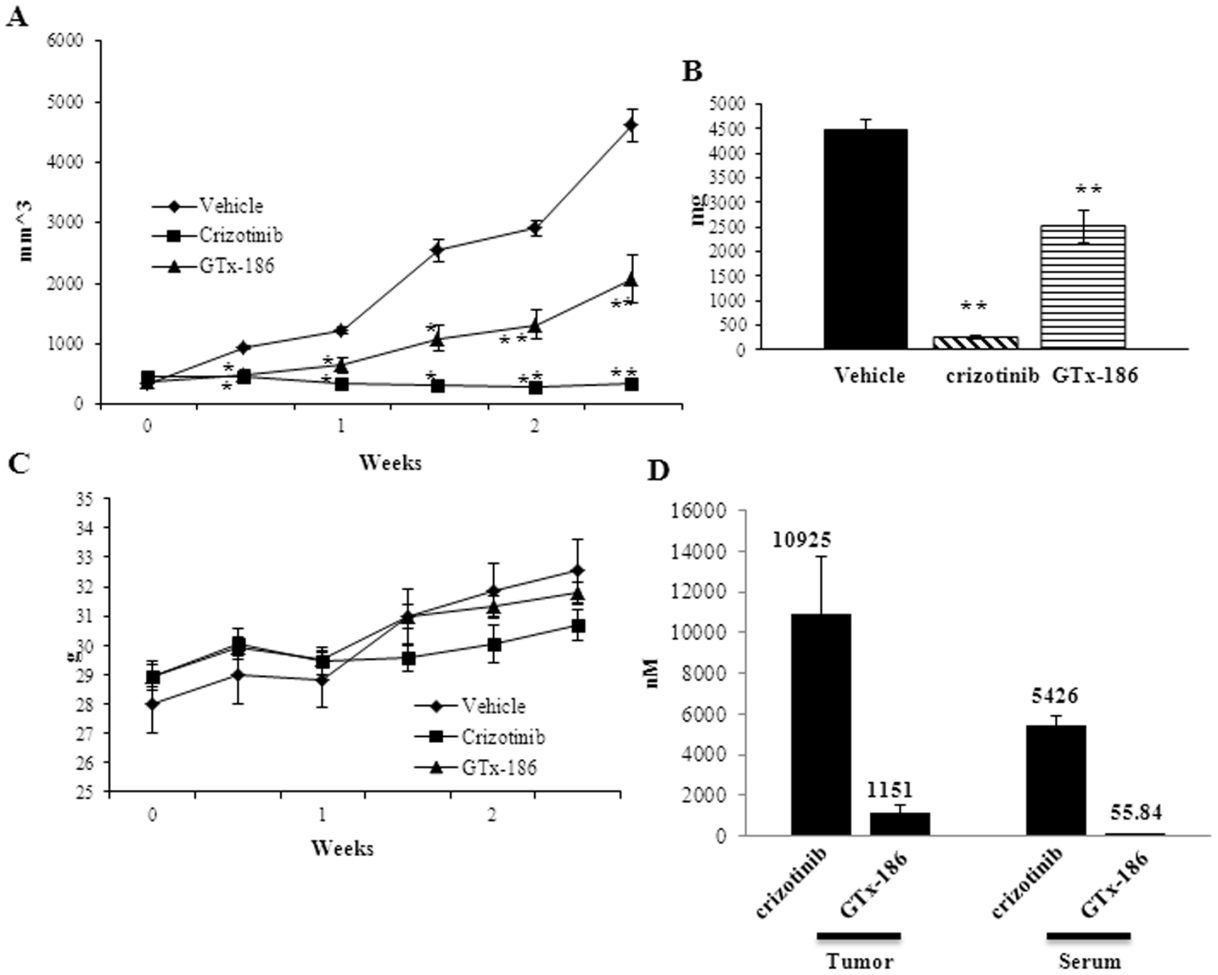
GTx-186 inhibits growth of NIH3T3-FIG-ROS1 xenograft. NIH3T3 cells stably over-expressing FIG-ROS1 were implanted subcutaneously in nude mice (2 million cells/mouse). Once tumors reached 100–200 mm^3^, the animals were randomized and treated daily with vehicle, 20 mg/kg/day GTx-186 i.v., or 75 mg/kg p.o. crizotinib. Tumor volumes (A) and body weight (C) were measured biweekly. After sacrifice, tumor weights (B) were recorded and the amount of drug in serum and tumors were quantified using LC-MS/MS (D). *-significant at p<0.01; **-significant at p<0.001. Values are expressed as Avg ± S.E. of n = 8.

To evaluate the effects of GTx-186 on ROS1-dependent tumor xenografts, NIH3T3:FIG-ROS1 (S) cells were implanted in nude mice. The animals were randomized when tumors reached 100–200 mm^3^ and treated with vehicle, GTx-186, or crizotinib. Both GTx-186 and crizotinib significantly reduced NIH3T3:FIG-ROS1 (S) tumor growth (Figure 7A) and tumor weight (Figure 7B), without affecting body weight (Figure 7C) or eliciting any visible toxicity. Though both GTx-186- and crizotinib-treated tumors were significantly smaller compared to vehicle-treated tumors, crizotinib performed better than GTx-186 *in vivo* resulting in 90% tumor growth inhibition.

To explain the apparent superiority of crizotinib over GTx-186 in the NIH3T3:FIG-ROS1 (S) xenografts, despite comparable *in vitro* potency, drug concentrations were measured in the tumors and serum collected five hours after the last dose (Figure 7D). Both local and systemic concentrations of crizotinib were 10- and 100-fold more, respectively, than GTx-186; indicating differences in pharmacokinetic properties could explain differences in tumor growth inhibition.

## Discussion

Here we synthesized and preclinically characterized novel RTK inhibitors that potently inhibited a subset of kinases important for the development of cancer and inflammation. RTKs have an extracellular ligand binding domain, a transmembrane hinge, and an intracellular cytoplasmic ATP binding pocket. The ATP binding pocket is highly conserved between RTKs, especially within the class belonging to the same phylogenetic branch. Most RTK inhibitors are developed to competitively bind to the ATP binding pocket, such that domain conservation between the members complicates attaining selectivity in RTK inhibitors. Similar to GTx-186, crizotinib, which was initially developed as a c-Met inhibitor, inhibits ALK and ROS1 equipotently. Considering that cancers find escape routes to overcome the selective pressure by activating alternate RTKs or secondary signaling, it follows that rational development of multi- inhibitors is a viable treatment strategy. Cancers overexpressing TRK-A and ROS1, such as those shown in Figure 1, could utilize both these proto-oncogenes to grow exponentially and metastasize. Similarly, lung cancers frequently overexpress ALK and ROS1, which may be inhibited by GTx-186 or crizotinib.

TRK-A is a potential therapeutic target with benefits for multiple indications. GTx-186 efficiently inhibited the following: neuroblastoma growth, localized inflammation such as dermatitis, systemic inflammation, and pain signaling. While GTx-186 inhibited inflammatory signaling mediated by AP-1 (PMA), NFκB (TNF-α), and TRK-A (NGF), dexamethasone inhibited only AP-1 and NFκB-mediated inflammation (Figure 3A). Interestingly, one of the factors up-regulated by croton oil was NGF, which was inhibited by GTx-186. This is not the first study to show increased NGF in inflammation. Earlier studies in chemically-induced inflammation of lung [46] and bladder [47] in mice and allergic inflammation [48] in humans had up-regulated serum NGF, which was considered the primary mediator of inflammation.

Similarly, TNF-α, an important mediator of air-pouch and other inflammation, was completely inhibited by GTx-186 (Figure 5C). CINCs or C-X-C chemokines, potent neutrophil chemoattractants implicated in neutrophil influx to acute inflammatory sites [49], were effectively inhibited by GTx-186, but not by indomethacin (Figure 5D). These results indicate that TRK-A inhibitors have anti-inflammatory effects distinct from glucocorticoids and COX inhibitors and of broader scope. The importance of chemokines as a paracrine factor in the epithelial:mesenchymal stem cell interaction and subsequent tumor metastasis has been well established [50]. Inhibition of these chemokines by GTx-186 will provide additional benefits to reduce tumor metastasis.

GTx-186 inhibited ROS1 at picomolar concentrations making it one of the most potent ROS1 inhibitors. In addition, GTx kinase inhibitors potently inhibited ALK *in vitro* kinase assays. We tested their effects on the phosphorylation of ALK and ALK-driven tumor cell growth. K-299 and SUDHL, two anaplastic large cell lymphoma (ALCL) cell lines that express NPM-ALK and are dependent on ALK for growth, were used to evaluate the efficacy of GTx-186 and crizotinib. GTx-186 and crizotinib comparably inhibited the growth of these two ALK (+) cell lines and ALK phosphorylation, but not the growth of ALK (−) U937 lymphoma cell line (Figure S2A and S2B). Inhibition of TRK, ROS, and ALK kinase activities makes GTx-186 a unique molecule.

There are six hallmarks of cancer including sustained proliferative signaling, resisting cell death, inducing angiogenesis, enabling replicative immortality, evading growth suppressors, and activating invasion and metastasis [51]. Inflammatory signaling is one of the newly recognized hallmarks of cancer that plays a role as important as the other six hallmarks. GTx-186 with its global anti-inflammatory effects and targeted anti-proliferative and pro-apoptotic effects, could provide sustained therapeutic benefit for cancers driven by its therapeutic targets.

Collectively, these studies underline the importance of ROS1 and TRK-A in promoting cancer and inflammatory diseases and further demonstrate the potential therapeutic utility of limited kinase cross-reactivity. Our findings suggest GTx-186 could become a valuable tool in the armamentarium to combat these diseases.

## Supporting Information

Figure S1 **GTx-186 inhibits neurite outgrowth and pain response early genes in PC12 pheochromocytoma cells.** A. PC12 cells were grown in 2% FBS, pre-treated with vehicle or 100 nM GTx-186 for 30 min and treated with vehicle or 100 ng/ml NGF for 7 days (with medium change and re-treatment on day 3). On day 7, cells were fixed, stained with sulforhodamine blue (SRB) and neurite outgrowth imaged using light microscope. B. GTx-186 does not inhibit proliferation of PC12 cells. PC12 neuroblastoma cells were plated and treated as indicated above for 7 days. Cells were fixed and stained with sulforhodamine blue (SRB) and optical density (OD) was measured at 535 nm. C. GTx-186 inhibits NGF-induced gene expression. PC12 cells were serum starved for 3 days and were pre-treated with indicated concentrations of GTx-186 for 30 min and treated with NGF or EGF for 45 minutes. RNA was extracted and the expression of genes was measured and normalized to GAPDH on a realtime rtPCR using TaqMan primers and probes. Values are expressed as Average ± S.E. of n = 3.
(PPTX)

Figure S2 **GTx-186 is a potent inhibitor of ALK-phosphorylation and ALK-dependent ALCL growth.** A. GTx-186 inhibits anaplastic large cell leukemia (ALCL) cell growth. Two ALK(+) ALCL lines (K-299, SUDHL-1) and an ALK(−) lymphoma line (U937) were treated with increasing concentrations of GTx-186 and crizotinib for 3 days. Cell growth was determined using WST-1, and IC_50_s values were determined and reported in nM. B. GTx-186 inhibits phosphorylation of ALK. K-299 cells were treated with increasing concentrations of GTx-186 or crizotinib for 4 hours. Protein lysates were the evaluated for p-ALK expression by ELISA.
(PPTX)

Table S1 **Specific activity and concentration of ATP and kinases used for kinase activity assays.**
(PPTX)

Table S2 **Cytokine array quantification.** Spots in cytokine array shown in Figure 5D were quantified densitometrically and expressed as Average ± S.E. (n = 3).
(PPTX)
