# Supplementary material for: Discovery and Preclinical Characterization of Novel Small Molecule TRK and ROS1 Tyrosine Kinase Inhibitors for the Treatment of Cancer and Inflammation
Source: PLoS One. 2013 Dec 26;8(12):e83380. doi: 10.1371/journal.pone.0083380 (PMC3873281; doi:10.1371/journal.pone.0083380)
Supplement: Figure S1 — GTx-186 inhibits neurite outgrowth and pain response early genes in PC12 pheochromocytoma cells. A. PC12 cells were grown in 2% FBS, pre-treated with vehicle or 100 nM GTx-186 for 30 min and treated with vehicle or 100 ng/ml NGF for 7 days (with medium change and re-treatment on day 3). On day 7, cells were fixed, stained with sulforhodamine blue (SRB) and neurite outgrowth imaged using light microscope. B. GTx-186 does not inhibit proliferation of PC12 cells. PC12 neuroblastoma cells were plated and treated as indicated above for 7 days. Cells were fixed and stained with sulforhodamine blue (SRB) and optical density (OD) was measured at 535 nm. C. GTx-186 inhibits NGF-induced gene expression. PC12 cells were serum starved for 3 days and were pre-treated with indicated concentrations of GTx-186 for 30 min and treated with NGF or EGF for 45 minutes. RNA was extracted and the expression of genes was measured and normalized to GAPDH on a realtime rtPCR using TaqMan primers and probes. Values are expressed as Average ± S.E. of n = 3. (PPTX) [file pone.0083380.s001.pptx]

## Slide 1
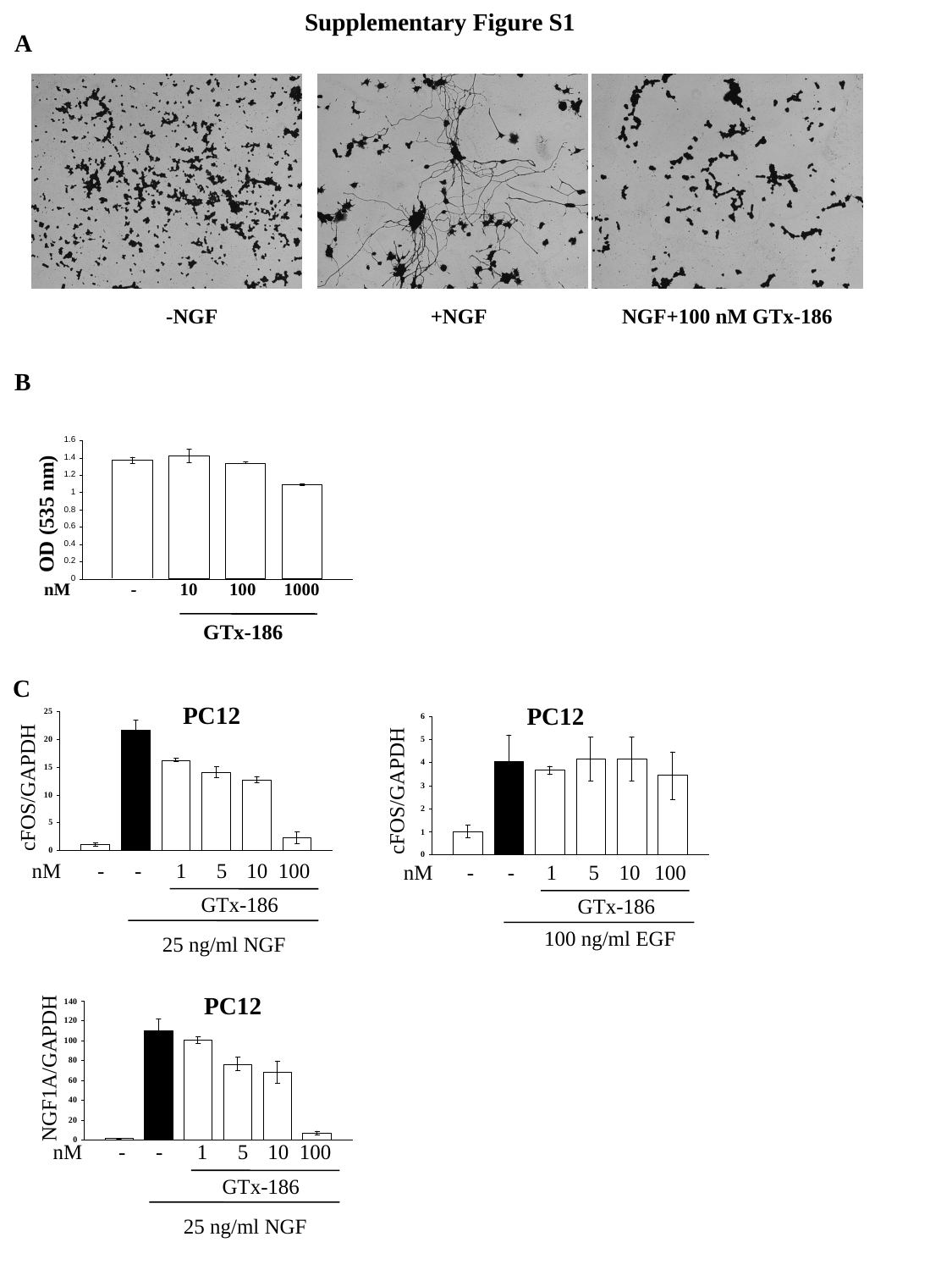

Supplementary Figure S1
A
-NGF
+NGF
NGF+100 nM GTx-186
B
OD (535 nm)
nM
-
10
100
1000
GTx-186
C
PC12
PC12
cFOS/GAPDH
cFOS/GAPDH
10
100
nM
-
-
1
5
10
100
nM
-
-
1
5
GTx-186
GTx-186
100 ng/ml EGF
25 ng/ml NGF
PC12
NGF1A/GAPDH
10
100
nM
-
-
1
5
GTx-186
25 ng/ml NGF
