# Supplementary figures and images for: Discovery and Preclinical Characterization of Novel Small Molecule TRK and ROS1 Tyrosine Kinase Inhibitors for the Treatment of Cancer and Inflammation
Source: PLoS One. 2013 Dec 26;8(12):e83380. doi: 10.1371/journal.pone.0083380 (PMC3873281; doi:10.1371/journal.pone.0083380)

## Slide 1
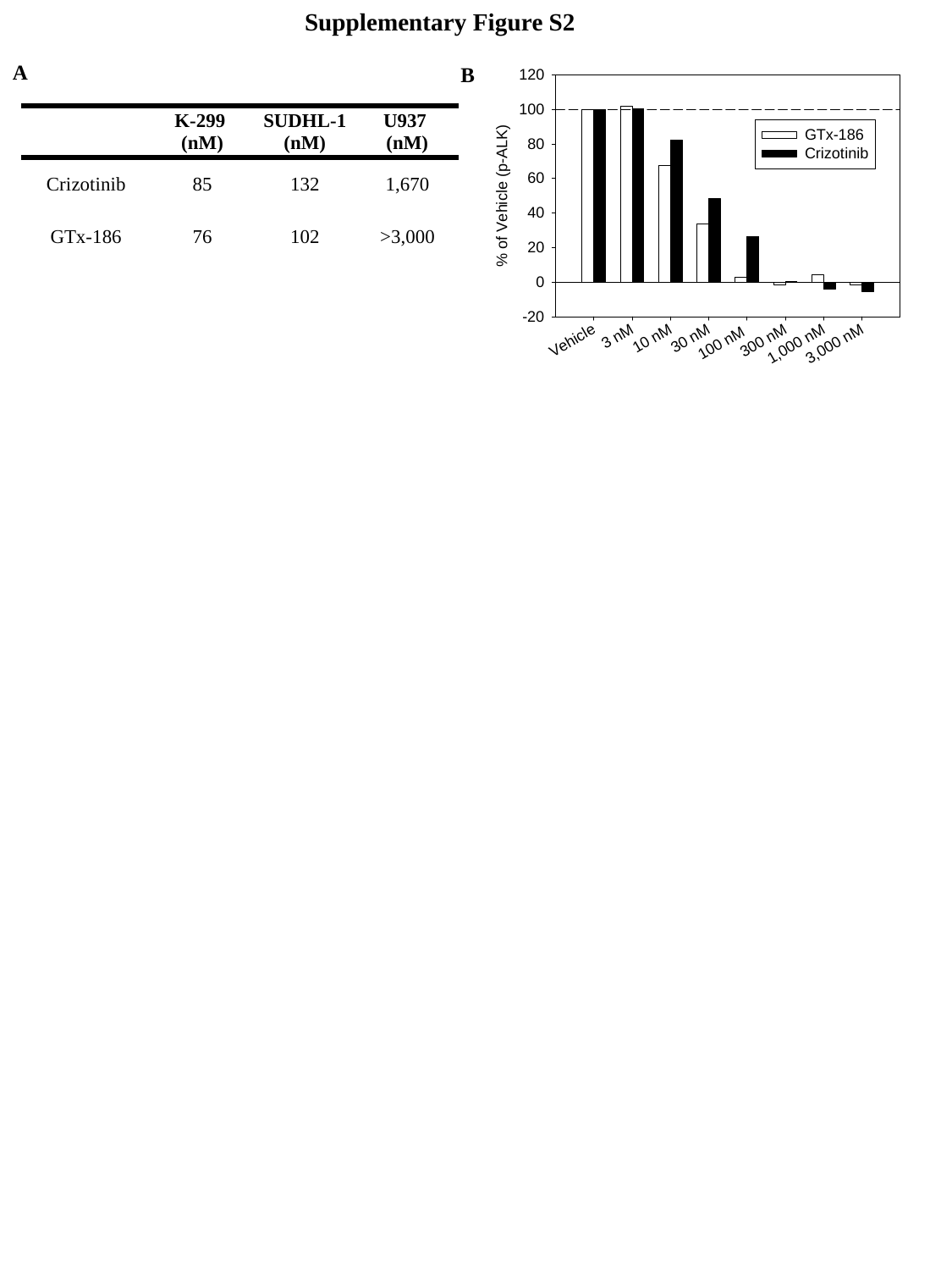

Supplementary Figure S2
A
B
| | K-299 (nM) | SUDHL-1 (nM) | U937 (nM) |
| --- | --- | --- | --- |
| Crizotinib | 85 | 132 | 1,670 |
| GTx-186 | 76 | 102 | >3,000 |

Supplement: Figure S2 — GTx-186 is a potent inhibitor of ALK-phosphorylation and ALK-dependent ALCL growth. A. GTx-186 inhibits anaplastic large cell leukemia (ALCL) cell growth. Two ALK(+) ALCL lines (K-299, SUDHL-1) and an ALK(−) lymphoma line (U937) were treated with increasing concentrations of GTx-186 and crizotinib for 3 days. Cell growth was determined using WST-1, and IC50s values were determined and reported in nM. B. GTx-186 inhibits phosphorylation of ALK. K-299 cells were treated with increasing concentrations of GTx-186 or crizotinib for 4 hours. Protein lysates were the evaluated for p-ALK expression by ELISA. (PPTX) [file pone.0083380.s002.pptx]
