# Supplementary material for: Discovery and Preclinical Characterization of Novel Small Molecule TRK and ROS1 Tyrosine Kinase Inhibitors for the Treatment of Cancer and Inflammation
Source: PLoS One. 2013 Dec 26;8(12):e83380. doi: 10.1371/journal.pone.0083380 (PMC3873281; doi:10.1371/journal.pone.0083380)
Supplement: Table S1 — Specific activity and concentration of ATP and kinases used for kinase activity assays. (PPTX) [file pone.0083380.s003.pptx]

## Slide 1
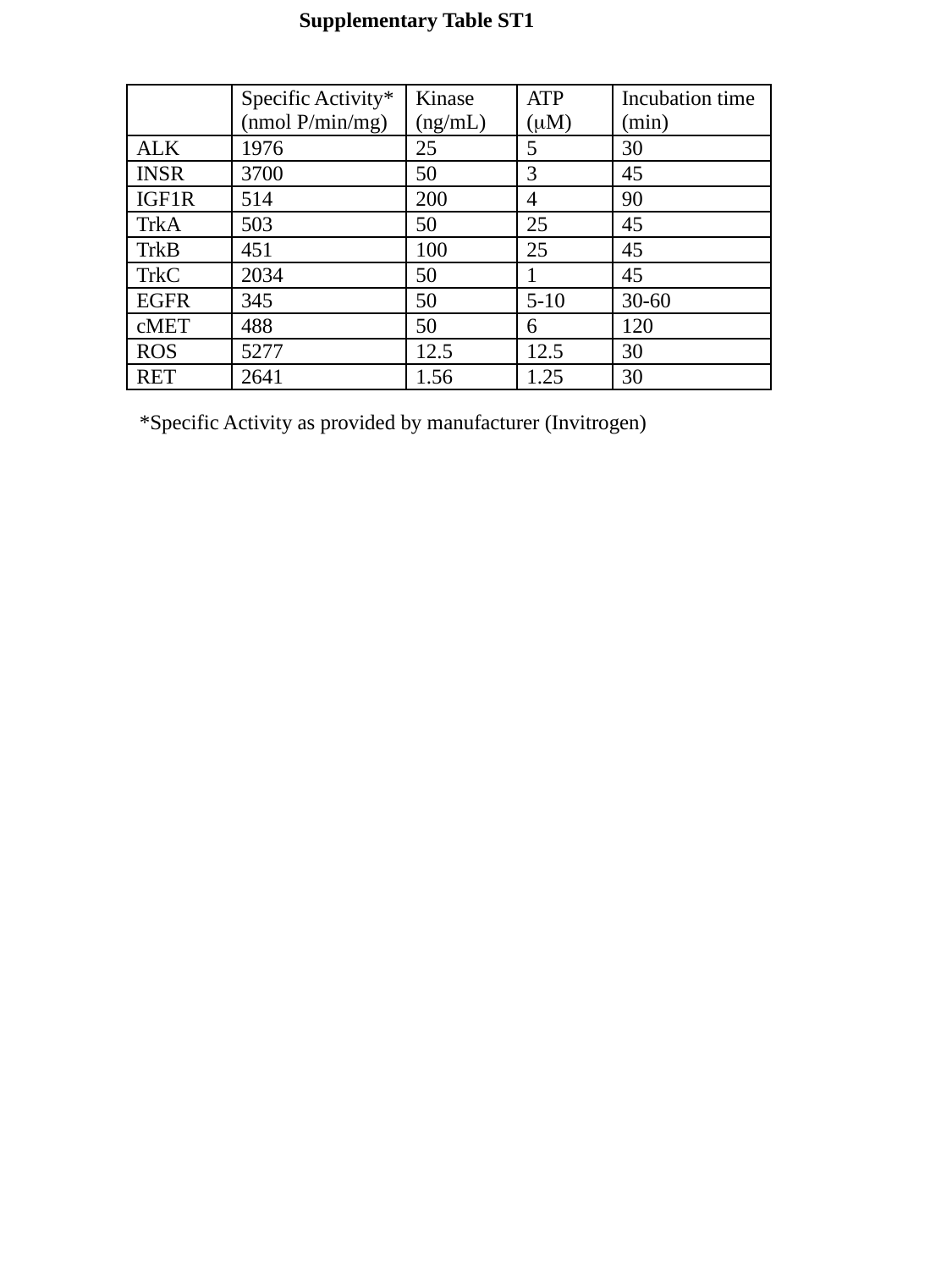

Supplementary Table ST1
| | Specific Activity\* (nmol P/min/mg) | Kinase (ng/mL) | ATP (μM) | Incubation time (min) |
| --- | --- | --- | --- | --- |
| ALK | 1976 | 25 | 5 | 30 |
| INSR | 3700 | 50 | 3 | 45 |
| IGF1R | 514 | 200 | 4 | 90 |
| TrkA | 503 | 50 | 25 | 45 |
| TrkB | 451 | 100 | 25 | 45 |
| TrkC | 2034 | 50 | 1 | 45 |
| EGFR | 345 | 50 | 5-10 | 30-60 |
| cMET | 488 | 50 | 6 | 120 |
| ROS | 5277 | 12.5 | 12.5 | 30 |
| RET | 2641 | 1.56 | 1.25 | 30 |
*Specific Activity as provided by manufacturer (Invitrogen)
