# Supplementary material for: Discovery and Preclinical Characterization of Novel Small Molecule TRK and ROS1 Tyrosine Kinase Inhibitors for the Treatment of Cancer and Inflammation
Source: PLoS One. 2013 Dec 26;8(12):e83380. doi: 10.1371/journal.pone.0083380 (PMC3873281; doi:10.1371/journal.pone.0083380)
Supplement: Table S2 — Cytokine array quantification. Spots in cytokine array shown in Figure 5D were quantified densitometrically and expressed as Average ± S.E. (n = 3). (PPTX) [file pone.0083380.s004.pptx]

## Slide 1
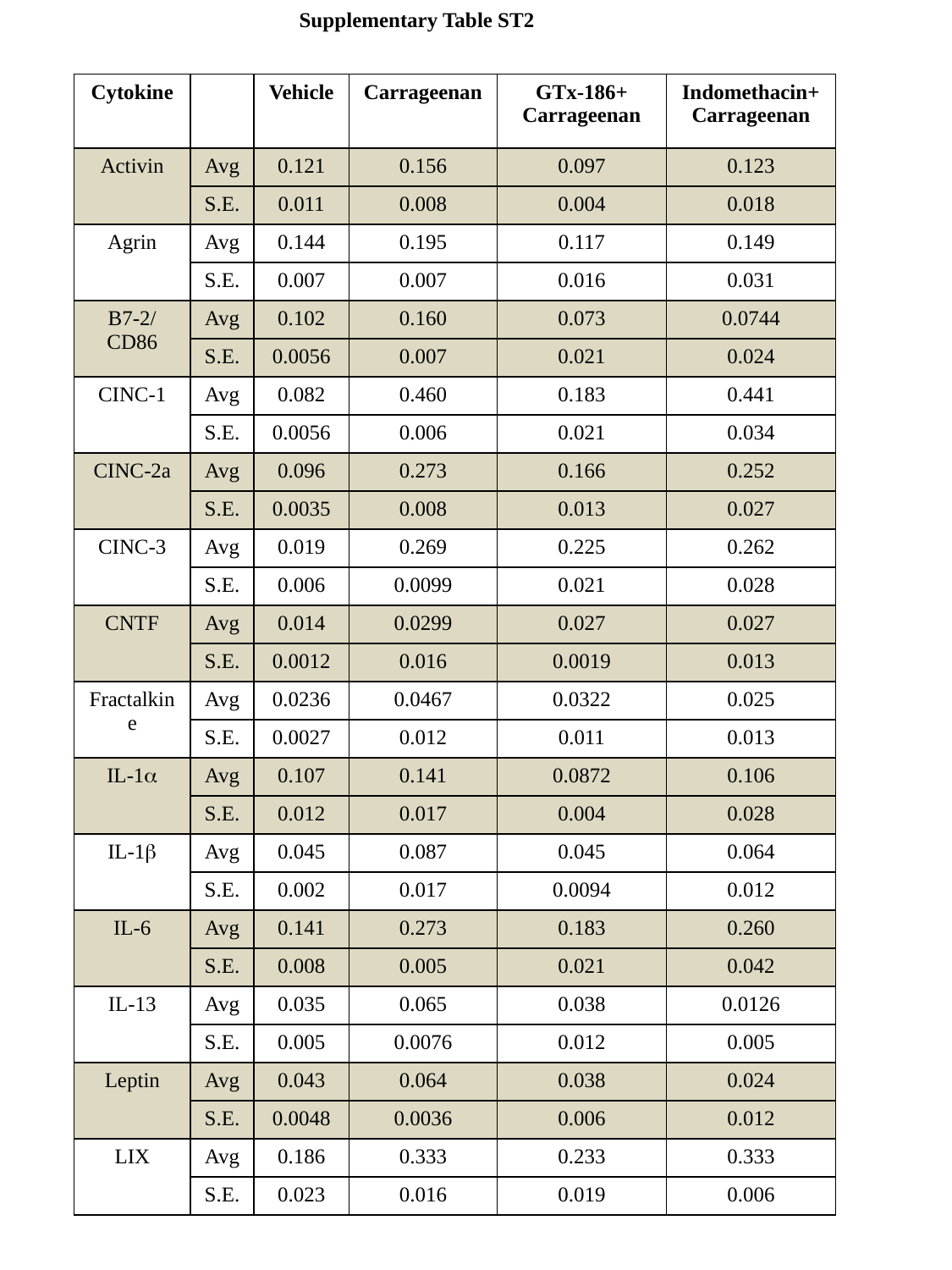

Supplementary Table ST2
| Cytokine | | Vehicle | Carrageenan | GTx-186+ Carrageenan | Indomethacin+Carrageenan |
| --- | --- | --- | --- | --- | --- |
| Activin | Avg | 0.121 | 0.156 | 0.097 | 0.123 |
| | S.E. | 0.011 | 0.008 | 0.004 | 0.018 |
| Agrin | Avg | 0.144 | 0.195 | 0.117 | 0.149 |
| | S.E. | 0.007 | 0.007 | 0.016 | 0.031 |
| B7-2/CD86 | Avg | 0.102 | 0.160 | 0.073 | 0.0744 |
| | S.E. | 0.0056 | 0.007 | 0.021 | 0.024 |
| CINC-1 | Avg | 0.082 | 0.460 | 0.183 | 0.441 |
| | S.E. | 0.0056 | 0.006 | 0.021 | 0.034 |
| CINC-2a | Avg | 0.096 | 0.273 | 0.166 | 0.252 |
| | S.E. | 0.0035 | 0.008 | 0.013 | 0.027 |
| CINC-3 | Avg | 0.019 | 0.269 | 0.225 | 0.262 |
| | S.E. | 0.006 | 0.0099 | 0.021 | 0.028 |
| CNTF | Avg | 0.014 | 0.0299 | 0.027 | 0.027 |
| | S.E. | 0.0012 | 0.016 | 0.0019 | 0.013 |
| Fractalkine | Avg | 0.0236 | 0.0467 | 0.0322 | 0.025 |
| | S.E. | 0.0027 | 0.012 | 0.011 | 0.013 |
| IL-1α | Avg | 0.107 | 0.141 | 0.0872 | 0.106 |
| | S.E. | 0.012 | 0.017 | 0.004 | 0.028 |
| IL-1β | Avg | 0.045 | 0.087 | 0.045 | 0.064 |
| | S.E. | 0.002 | 0.017 | 0.0094 | 0.012 |
| IL-6 | Avg | 0.141 | 0.273 | 0.183 | 0.260 |
| | S.E. | 0.008 | 0.005 | 0.021 | 0.042 |
| IL-13 | Avg | 0.035 | 0.065 | 0.038 | 0.0126 |
| | S.E. | 0.005 | 0.0076 | 0.012 | 0.005 |
| Leptin | Avg | 0.043 | 0.064 | 0.038 | 0.024 |
| | S.E. | 0.0048 | 0.0036 | 0.006 | 0.012 |
| LIX | Avg | 0.186 | 0.333 | 0.233 | 0.333 |
| | S.E. | 0.023 | 0.016 | 0.019 | 0.006 |

## Slide 2
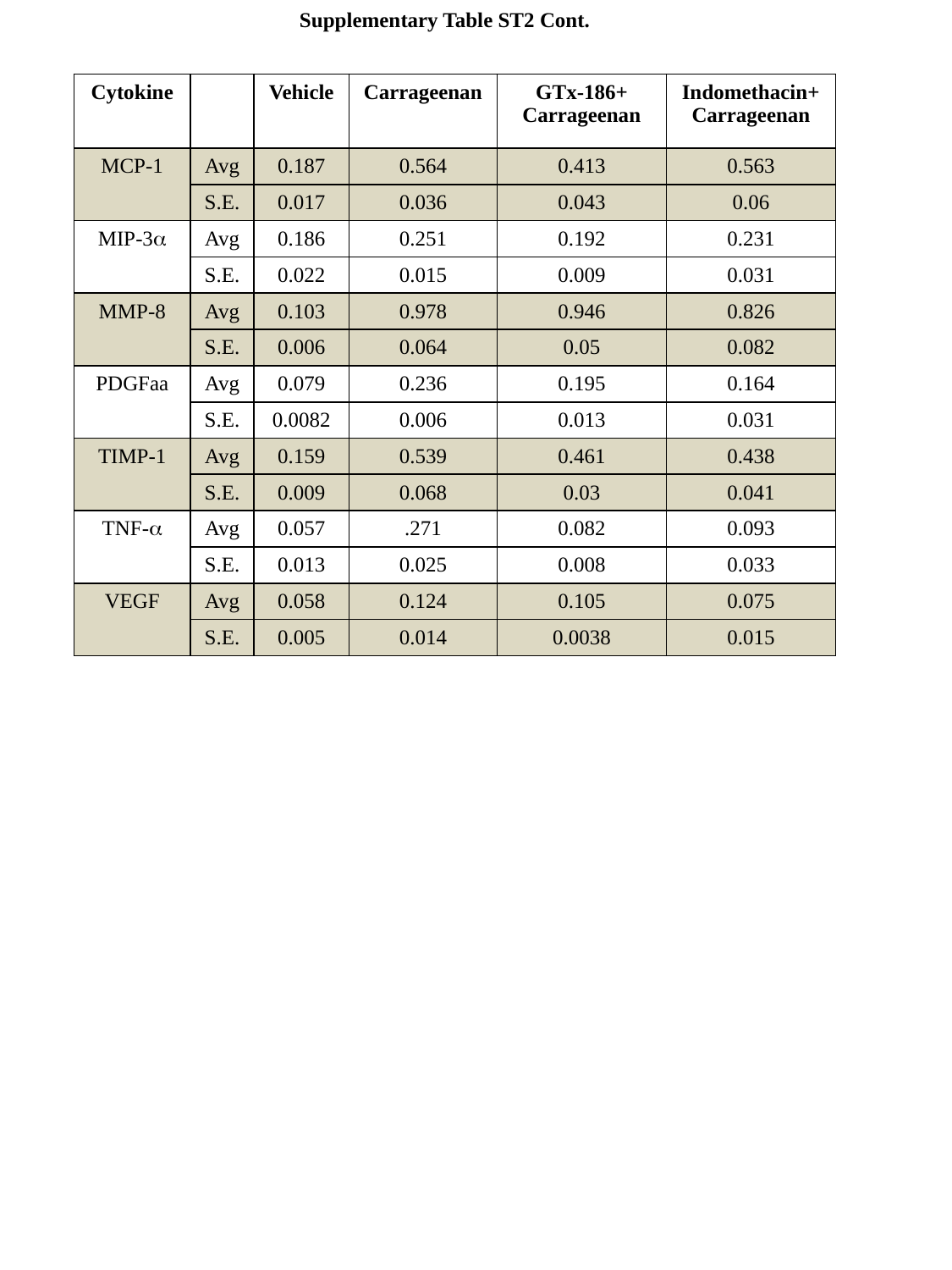

Supplementary Table ST2 Cont.
| Cytokine | | Vehicle | Carrageenan | GTx-186+ Carrageenan | Indomethacin+Carrageenan |
| --- | --- | --- | --- | --- | --- |
| MCP-1 | Avg | 0.187 | 0.564 | 0.413 | 0.563 |
| | S.E. | 0.017 | 0.036 | 0.043 | 0.06 |
| MIP-3α | Avg | 0.186 | 0.251 | 0.192 | 0.231 |
| | S.E. | 0.022 | 0.015 | 0.009 | 0.031 |
| MMP-8 | Avg | 0.103 | 0.978 | 0.946 | 0.826 |
| | S.E. | 0.006 | 0.064 | 0.05 | 0.082 |
| PDGFaa | Avg | 0.079 | 0.236 | 0.195 | 0.164 |
| | S.E. | 0.0082 | 0.006 | 0.013 | 0.031 |
| TIMP-1 | Avg | 0.159 | 0.539 | 0.461 | 0.438 |
| | S.E. | 0.009 | 0.068 | 0.03 | 0.041 |
| TNF-α | Avg | 0.057 | .271 | 0.082 | 0.093 |
| | S.E. | 0.013 | 0.025 | 0.008 | 0.033 |
| VEGF | Avg | 0.058 | 0.124 | 0.105 | 0.075 |
| | S.E. | 0.005 | 0.014 | 0.0038 | 0.015 |
